# Spike-Conducting Integrate-and-Fire Model

**DOI:** 10.1523/ENEURO.0112-18.2018

**Published:** 2018-09-07

**Authors:** Go Ashida, Waldo Nogueira

**Affiliations:** 1 Cluster of Excellence “Hearing4all,” Department of Neuroscience, Faculty 6, University of Oldenburg, 26129 Oldenburg, Germany; 2 Cluster of Excellence “Hearing4all,” Department of Otolaryngology, Hannover Medical School, 30625 Hannover, Germany

**Keywords:** action potential propagation, spike conduction, electrical stimulation, computational model, auditory nerve

## Abstract

Modeling is a useful tool for investigating various biophysical characteristics of neurons. Recent simulation studies of propagating action potentials (spike conduction) along axons include the investigation of neuronal activity evoked by electrical stimulation from implantable prosthetic devices. In contrast to point-neuron simulations, where a large variety of models are readily available, Hodgkin–Huxley-type conductance-based models have been almost the only option for simulating axonal spike conduction, as simpler models cannot faithfully replicate the waveforms of propagating spikes. Since the amount of available physiological data, especially in humans, is usually limited, calibration, and justification of the large number of parameters of a complex model is generally difficult. In addition, not all simulation studies of axons require detailed descriptions of nonlinear ionic dynamics. In this study, we construct a simple model of spike generation and conduction based on the exponential integrate-and-fire model, which can simulate the rapid growth of the membrane potential at spike initiation. In terms of the number of parameters and equations, this model is much more compact than conventional models, but can still reliably simulate spike conduction along myelinated and unmyelinated axons that are stimulated intracellularly or extracellularly. Our simulations of auditory nerve fibers with this new model suggest that, because of the difference in intrinsic membrane properties, the axonal spike conduction of high-frequency nerve fibers is faster than that of low-frequency fibers. The simple model developed in this study can serve as a computationally efficient alternative to more complex models for future studies, including simulations of neuroprosthetic devices.

## Significance Statement

Conduction of electrical impulses (action potentials) along the axon is essential for information transfer between neurons. Simulation studies of propagating action potentials, which earlier focused on the biophysical mechanisms of conduction, have progressed to investigations of pathologic malfunctions of nerves and electrical stimulations via prostheses. In contrast to dimensionless, single-neuron modeling, for which a number of different approaches are available, simulation of nerve conduction generally requires a complex model of ionic conductances to reproduce propagating action potentials. In this study, we present a simplified phenomenological model of axonal conduction with increased computational efficiency and a reduced number of parameters. This simple model can be used as an alternative to conventional models, especially for applications including prosthetic simulations of nerve conduction.

## Introduction

Since Louis Lapicque (Lapicque, 1907) first introduced its underlying idea of parallel capacitor and leak resistor in combination with threshold crossing, the integrate-and-fire (IF) model has served as a useful tool to simulate spiking activity of neuronal membranes. Even after the detailed ionic dynamics underlying spike generation were discovered and modeled with a more complex conductance-based description (Hodgkin and Huxley, 1952), the IF model and its variations are still frequently used as a convenient alternative to Hodgkin–Huxley (HH)-type models, especially when computational efficiency and mathematical transparency are required. Applications of the IF model include large-scale simulations of a neuronal network, and rigorous analysis of neuronal spiking responses driven by random synaptic inputs (for representative examples, see Koch, 1999; Gerstner et al., 2014).

Within the family of IF-type models, various nonlinear versions have been created (Gerstner et al., 2014). For instance, the exponential IF (EIF) model was introduced to describe the exponential growth of the membrane potential at spike initiation (Fourcaud-Trocmé et al., 2003). Its subthreshold response properties to stimulus current injections remain largely unchanged from the Wang–Buzsáki (WB) model, which itself is a modification of the HH model (Wang and Buzsáki, 1996). In the category of single-compartment models, a modified version of the EIF model was shown to replicate the rapid initiation of action potentials even better than more detailed HH-type models (Brette, 2015). Although the EIF model was originally created to simulate the spiking activity of cortical neurons, its variations are now used to simulate a wider range of cells [auditory nerve (AN) fibers: Rutherford et al., 2012; Joshi et al., 2017; visuomotor system: Morén et al., 2013; cerebellar Purkinje cell: Ostojic et al., 2015; optic nerves: Arancibia-Cárcamo et al., 2017].

In the domain of single-compartment models, several levels of abstraction are possible: from biophysical conductance-based descriptions equipped with a variety of ion channels, via IF-type models with intermediate complexity, to more phenomenological “black-box” approaches that focus solely on the input-output functions (Herz et al., 2006). This gradient of biological plausibility and computational efficiency enables a user to select an appropriate single-compartment model depending on the specific purpose of modeling (Ashida et al., 2017). In contrast, for multicompartment neuronal modeling, in which multiple nonlinear excitable units are connected with each other, HH-type models are normally the only options, since the abrupt reset of the membrane potential in an IF-type model is generally incompatible with spatially propagating electrical activity over the modeled membrane. Earlier studies using multicompartment spiking neuron models simulated the conduction of action potentials along the axon. For myelinated axons, for example, each node of Ranvier was modeled as a HH-type excitable compartment that was interconnected with an axial resistance (FitzHugh, 1962; Brill et al., 1977; Moore et al., 1978). For unmyelinated axons, the HH model was combined with the cable equation to account for the spatial extension of the axon (Cooley and Dodge, 1966). Compartmental models of spike conduction were later applied to simulate, for example, pathologic changes of axons (Coggan et al., 2010; Brown and Hamann, 2014) and the interaction between nerves and prosthetic devices (for a review, see Rattay et al., 2003).

Despite the general success of HH-type models in reproducing axonal spike conduction, not all simulation studies actually require the detailed descriptions of ion channel dynamics. Moreover, neurophysiological data from humans, in particular for single-cell properties, are usually sparse, making it difficult to calibrate or justify the parameters of a model used for prosthetic nerve simulations. Recent prosthetic modeling aims to simulate tens of nodes in thousands of nerves distributed three-dimensionally (Nogueira et al., 2016), which requires the efficient model representation of excitable units. In this study, we propose a simple model of action-potential propagation along the axon based on the EIF model of spike generation. The model has much fewer parameters than the HH model but still faithfully reproduces axonal spike conduction. As an example application, we fit the model to known physiological data from ANs. Our simulated conduction velocities match the experimentally measured range in AN fibers, confirming the applicability of the model. We expect that the model introduced in this study will serve as a simpler replacement for the HH model especially when computational performance and structural simplicity are preferred over the biophysical details of spike generation.

## Materials and Methods

### Overview of the three models

In this article, we compare three types of spiking membrane models: (1) the WB model, a variation of the HH model, having nonlinear sodium and potassium conductances (Wang and Buzsáki, 1996); (2) the original version of the EIF model (Fourcaud-Trocmé et al., 2003), which we refer to as the standard EIF (sEIF) model; and (3) a modified version of the EIF model, called the bounded EIF (bEIF) model, with a “ceiling” for the spike-generating, depolarizing current. The sEIF model was originally created (and fitted) to study the spike generation of the WB model (Fourcaud-Trocmé et al., 2003), and we here introduce the bEIF model as a modification of the sEIF model to account for axonal spike conduction.

All these models share an equation of the form:

(1) $$C_{m}\frac{\text{d}}{\text{dt}}V(t)={\text{G}}_{\text{L}}({\text{E}}_{\text{L}}-V)+\psi (V)+I_{\text{inj}},$$

where *C_m_* is the membrane capacitance, G_L_ is the time- and voltage-independent (linear) leak conductance, E_L_ is the leak reversal potential, *I*_inj_ is the intracellularly injected current, and *ψ*(*V*) is a nonlinear function of the membrane potential *V* responsible for spike generation. In the WB model, *ψ*(*V*) is a sum of sodium *I*_Na_ and potassium *I*_K_ currents:

(2a) $$\psi (V)=I_{Na}+I_{K}.$$

The activation/inactivation kinetics of these currents are described by three additional differential equations (Table 1). In the sEIF model, *ψ*(*V*) is equal to the depolarizing current *I*_dep_:

(2b) $$\psi (V)=I_{dep},$$

which is an exponential function of the membrane potential *V* describing the exponential growth of the membrane potential at spike initiation (Table 2). The after-spike repolarization in the sEIF model is simply a reset of *V* to the resting membrane potential. In the bEIF model, *ψ*(*V*) is a sum of depolarizing *I*_dep_ and repolarizing *I*_rep_ currents:

(2c) $$\psi (V)=I_{\mathrm{dep}}+I_{\mathrm{rep}}.$$

**Table 1.** Equations and parameters for the single-compartment WB model

**Variable**	**Equation**
Membrane potential *V*	$C_{m}\;dV(t)/dt=I_{\text{L}}+I_{\text{K}}+I_{\text{Na}}+I_{\text{inj}}$
Leak current	$I_{\text{L}}={\text{G}}_{\text{L}}\cdot ({\text{E}}_{\text{L}}-V)$
Delayed rectifier K current	$I_{\text{K}}={\text{G}}_{\text{K}}\cdot n^{4}\cdot ({\text{E}}_{\text{K}}-V)$
Fast (transient) Na current	$I_{\text{Na}}={\text{G}}_{\text{Na}}\cdot m^{3}h\cdot ({\text{E}}_{\text{Na}}-V)$
Intracellularly injected current	$I_{\text{inj}}=0$ (default)
Kinetic equations for channel variables (*y* = *m*, *h*, or *n*)	$dy(t)/dt=\alpha_{y}(V)(1-y)-\beta_{y}(V)\cdot y$
Rate functions for K activation *n*	$\alpha_{n}(V)=\frac{0.05\;(V+34)}{\left(1-\exp(-(V+34)/10)\right)}$ $\beta_{n}(V)=0.625\;\exp(-(V+44)/80)$
Rate functions for Na activation *m*	$\alpha_{m}(V)=\frac{0.50\;(V+35)}{\left(1-\exp(-(V+35)/10)\right)}$ $\beta_{m}(V)=20.0\;\exp(-(V+60)/18)$
Rate functions for Na inactivation *h*	$\alpha_{h}(V)=0.35\;\exp(-(V+58)/20)$ $\beta_{h}(V)=\frac{5.0}{\left(1+\exp(-(V+28)/10)\right)}$
**Parameter**	**Value**
Membrane capacitance density *C_*m*_*	1.0 μF/cm^2^
Leak conductance density G_L_	0.1 mS/cm^2^
K conductance density G_K_	15.0 mS/cm^2^
Na conductance density G_Na_	35.0 mS/cm^2^
Leak reversal potential E_L_	-65 mV
K reversal potential E_K_	-90 mV
Na reversal potential E_Na_	+55 mV

**Table 2.** Equations and parameters for the single-compartment sEIF model

**Variable**	**Equation**
Membrane potential *V*	$C_{m}\;dV(t)/dt=I_{\text{L}}+I_{\text{dep}}+I_{\text{inj}}$
Leak current	$I_{\text{L}}={\text{G}}_{\text{L}}\cdot ({\text{E}}_{\text{L}}-V)$
Spike-generating (depolarizing) current	$I_{\text{dep}}={\text{G}}_{\text{L}}K_{\text{T}}\;\exp((V-V_{\text{T}})/K_{\text{T}})$
Potential reset after spiking	$V(t_{+})\to V_{\text{reset}}$ when $V(t_{-})\geq V_{\text{spike}}$
Intracellularly injected current	$I_{\text{inj}}=0$ (default)
**Parameter**	**Value**
Membrane capacitance density *C_*m*_*	1.0 μF/cm^2^
Leak conductance density G_L_	0.1 mS/cm^2^
Leak reversal potential E_L_	-65.3 mV
Threshold for spike-generating current *V*_T_	-60.2 mV
Slope factor of the spike-generating current K_T_	3.5 mV
Spike-detecting threshold *V*_spike_	+15 mV
Reset potential *V*_reset_	-65.3 mV (same as E_L_)
Refractory period τ_ref_	2.8 ms

The depolarizing current *I*_dep_ represents the exponentially growing, inward current for spike initiation, while the repolarizing current *I*_rep_ corresponds to the outward current responsible for after-spike repolarization (Table 3). More detailed descriptions of each model are provided below. As in the previous work, in which the sEIF model was first introduced (Fourcaud-Trocmé et al., 2003), we use the WB model as a reference and compare its responses with those of the EIF models.

**Table 3.** Equations and parameters for the single-compartment bEIF model

**Variable**	**Equation**
Membrane potential *V*	$C_{m}\;dV(t)/dt=I_{\text{L}}+I_{\text{dep}}+I_{\text{rep}}+I_{\text{inj}}$
Leak current	$I_{\text{L}}={\text{G}}_{\text{L}}\cdot ({\text{E}}_{\text{L}}-V)$
Spike-generating (depolarizing) current	$I_{\text{dep}}={\text{G}}_{\text{L}}K_{\text{T}}\frac{A_{T}}{\left(1+A_{T}\;\exp(-(V-V_{T})/K_{\text{T}})\right)}$
Repolarizing current	$I_{\text{rep}}={\text{G}}_{\text{rep}}(t)\cdot ({\text{E}}_{\text{L}}-V)$
Starting time of repolarizing current	${\text{T}}_{\text{rep}}:=t$ when $V(t)\geq V_{\text{rep}}$
Repolarizing conductance (for *t* ≥ T_rep_)	${\text{G}}_{\text{rep}}(t)={\text{G}}_{\text{L}}A_{\text{rep}}\left(\frac{\left(t-{\text{T}}_{\text{rep}}\right)}{\tau_{\text{rep}}}\right)\exp\left(1-\frac{\left(t-{\text{T}}_{\text{rep}}\right)}{\tau_{\text{rep}}}\right)$
Intracellularly injected current	$I_{\text{inj}}=0$ (default)
**Parameter**	**Value**
Membrane capacitance density *C_*m*_*	1.0 μF/cm^2^
Leak conductance density G_L_	0.1 mS/cm^2^
Leak reversal potential E_L_	-65.3 mV
Threshold for spike-generating current *V*_T_	-60.2 mV
Slope factor of the spike-generating current *K*_T_	3.5 mV
Ceiling factor of the spike-generating current *A*_T_	520 (no unit)
Starting voltage of repolarization current *V*_rep_	+10 mV
Time constant of repolarizing conductance *τ*_rep_	0.60 ms
Amplitude factor of repolarizing conductance *A*_rep_	90 (no unit)

### Wang-Buzsáki model

The WB model is a set of nonlinear differential equations that describe the dynamics of the membrane potential *V*(*t*), the activation *m*(*t*) and inactivation *h*(*t*) of sodium channels, and the activation *n*(*t*) of potassium channels (Table 1). While in the original work of Wang and Buzsáki (1996), the sodium activation was assumed to be instantaneous, here we adopted a voltage-dependent time constant for sodium activation as in the original HH model (Hodgkin and Huxley, 1952). The resulting differences between instantaneous and time-delayed sodium activations are generally minor and limited to high-frequency sinusoidal input currents (Fourcaud-Trocmé et al., 2003). The membrane parameters we used (Table 1) were taken from Wang and Buzsáki (1996).

The single-compartment WB model has seven membrane parameters: one membrane capacitance (*C_m_*), three conductances (G_L_, G_K_, G_Na_), and three reversal potentials (E_L_, E_K_, E_Na_). In addition, each rate function for channel activation and inactivation (*α_m_*, *β_m_*, *α_h_*, *β_h_*, *α_n_*, *β_n_*) requires three parameters (amplitude, reference voltage, and slope factor). In total, the WB model needs 25 parameters to be calibrated to fit physiological data.

### Standard exponential integrate-and-fire model

The sEIF model, which is a nonlinear modification of the leaky (linear) IF model, phenomenologically describes the exponentially increasing sodium inward current at spike initiation (Fourcaud-Trocmé et al., 2003). Its spike-generating depolarizing current,

(3) $$I_{\text{dep}}={\text{G}}_{\text{L}}K_{\text{T}}\;\exp((V-V_{\text{T}})/K_{\text{T}}),$$

is characterized by the (soft) threshold *V*_T_ and the slope factor *K*_T_, which determine the excitability of the model neuron. Once the membrane potential *V* crosses the spike-detection threshold *V*_spike_, it is reset to and held at the resting potential *V*_reset_ for the refractory period τ_ref_.

**Figure 1.**
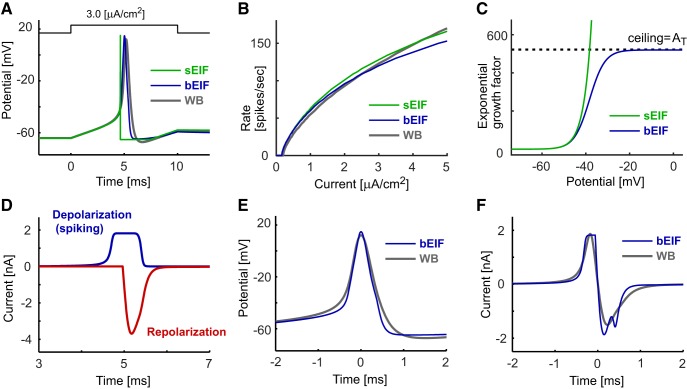
Response properties of single-compartment models. ***A***, Spike responses of the sEIF (green), bEIF (blue), and WB (gray) models driven by a step current input. ***B***, f-I curves of the models. Step currents of varied amplitudes were injected and the numbers of spikes in 1000 ms were calculated. ***C***, Voltage dependence of the exponential growth factors of the sEIF and bEIF models. ***D***, Depolarizing and repolarizing spike currents of the bEIF model. The horizontal corresponds to the expanded time in ***A***. ***E***, Spike shapes of the bEIF and WB models. ***F***, Membrane currents of the bEIF and WB models. In ***E***, ***F***, traces are aligned such that time 0 corresponds to the peak timings of the action potentials shown in ***A***.

The single-compartment sEIF model has eight parameters (Table 2). Since the spiking voltage of the sEIF model quickly diverges to infinity in a finite amount of time, the spike-detection threshold *V*_spike_ does not play a major role in determining the response property of the model (Touboul, 2009), thus reducing the effective number of unconstrained parameters to seven. As in the original sEIF study (Fourcaud-Trocmé et al., 2003), the parameters of the sEIF model in our study were selected so that the initial part of its spike waveform, including the subthreshold response (Fig. 1*A*), its spiking threshold, and its frequency-current (f-I) relationship resembled those of the WB model (Fig. 1*B*).

### Bounded exponential integrate-and-fire model

As we will see in the Results, the behavior of the sEIF membrane potential diverging to infinity (Fig. 1*A*) is incompatible with spike propagation along the axon. Hence, we replaced the exponential growth term exp((*V*-*V*_T_)/*K*_T_) in the sEIF model (Eq. 3) with *A*_T_/(1+*A*_T_ exp(-(*V*-*V*_T_)/*K*_T_)) to set a ceiling *A*_T_ for the inward current (Fig. 1*C*). Thus, in this modified model, named the bEIF here, the spike-generating current is written as:

(4) $$I_{\text{dep}}={\text{G}}_{\text{L}}K_{\text{T}}A_{T}/(1+A_{T}\exp(-(V-V_{\text{T}})/K_{\text{T}})),$$

and is bounded as: max(*I*_dep_) = G_L_*K*_T_*A*_T_. This modification resulted in a slower (and probably more realistic) voltage increase near the peak of an action potential while keeping the sub- and near-threshold responses almost identical to the sEIF model (Fig. 1*A*).

In contrast to the instantaneous potential reset of the sEIF model, the bEIF model has an additional repolarizing current *I*_rep_ to mimic the downward trajectory of the membrane potential after each spike (Fig 1*A*; for the equations, see Table 3). This current is initiated when the potential *V* reaches the preset starting voltage *V*_rep_, rapidly overwhelming the depolarization current (Fig. 1*D*) to bring the membrane potential back to the resting level (Fig. 1*A*). We used an alpha function for the repolarizing conductance, since it allows fast and exact calculation at each time step (Rotter and Diesmann, 1999). Simulated spike shapes were similar between the single-compartment WB and bEIF models, except for the slightly narrower spike width and the lack of after-spike hyperpolarization in the bEIF model (Fig. 1*E*). The total membrane currents during a spike are also comparable between these two models, both in amplitude and time course (Fig. 1*F*).

The bEIF model has nine parameters (Table 3): three for subthreshold responses (*C_m_*, G_L_, and E_L_), another three for spiking (*V*_T_, *K*_T_, and *A*_T_), and the remaining three for repolarization (*V*_rep_, *τ*_rep_, and *A*_rep_). In this study, the ceiling factor *A*_T_ and three repolarization parameters of the bEIF model were adjusted to mimic the spike shape (Fig. 1*A*) and f-I curve (Fig. 1*B*) of the WB model, while the other five parameters were unchanged from the sEIF model. It should be noted that, unlike the sEIF model, the bEIF model does not have an explicit refractory period as a model parameter, because the repolarizing current *I*_rep_, which rapidly overcomes the spike current *I*_dep_, effectively suppresses spike generation for a certain time period. The length of this “dead time” is determined by the shape (amplitude and time scale) of the repolarizing current.

### Simulating myelinated axons

Myelinated axons were modeled as a series of excitable units interconnected with an axial resistance (Table 4). The excitability of each nodal compartment is described either by the WB model or the bEIF model. For simplicity, we considered the ideal situation, in which the myelinated internodes are perfectly insulated (i.e., with negligible capacitance and transmembrane conductance; McNeal, 1976; Keener and Sneyd, 2009), although simulations suggested imperfect insulation might affect both excitability and conduction (McIntyre et al., 2002; Young et al., 2013). Furthermore, we also simply assumed that all ion channels (of the WB model) are located at the nodes of Ranvier, despite the accumulating evidence of non-uniform distribution of ion channels at and around the node (Hossain et al., 2005; Yi et al., 2010; Kim and Rutherford, 2016; for reviews, see Salzer et al., 2008; Debanne et al., 2011; Freeman et al., 2016). The default parameter values of our myelinated axon model are shown in Table 4. For each excitable node, we used the same WB (Table 1) or bEIF (Table 3) description as for the single-compartment model.

**Table 4.** Equations and parameters for myelinated axon models

**Variable**	**Equation**
Membrane potential *V* (of j-th node)	$c_{m}^{j}\;dV_{j}(t)/dt=I_{\text{L}}^{j}+I_{\text{active}}^{j}+I_{\text{axon}}^{j}+I_{\text{inj}}^{j}$
Leak current	$I_{\text{L}}^{j}={\text{g}}_{\text{L}}^{j}(E_{\text{L}}-V_{j})$
Active current (for WB model)	$I_{\text{active}}^{j}=I_{\text{Na}}^{j}+I_{\text{K}}^{j}$
Active current (for bEIF model)	$I_{\text{active}}^{j}=I_{\text{dep}}^{j}+I_{\text{rep}}^{j}$
Axonal current	$I_{\text{axon}}^{j}={\text{g}}_{\text{axon}}^{j-1}(V_{j-1}-V_{j})+{\text{g}}_{\text{axon}}^{j}(V_{j+1}-V_{j})$
Intracellularly injected current	$I_{\text{inj}}^{j}=0$ (default)
Membrane capacitance	$c_{m}^{j}=\pi DL_{n}C_{m}$
Leak conductance	${\text{g}}_{\text{L}}^{j}=\pi DL_{n}{\text{G}}_{\text{L}}$
Axonal resistance	$r_{\text{axon}}^{j}=\frac{4L_{i}R_{\text{ax}}}{\pi D^{2}}$
Axonal conductance	${\text{g}}_{\text{axon}}^{j}=1/R_{\text{axon}}^{j}=\frac{\pi D^{2}}{4L_{i}R_{\text{ax}}}$
**Parameter**	**Value**
Axon diameter *D*	2 μm
Nodal length *L_*n*_*	2 μm
Internodal length *L_*i*_*	200 μm
Axial resistivity R_ax_	100 Ω cm

Parameters not listed in this table are unchanged from the single-compartment models (Tables 1, 3).

We simulated 141 nodes along a one-dimensional (non-branching) axon. Stimulus currents (amplitude *I*_inj_ = 100 pA, duration T = 1 ms) were injected intracellularly into the node #20 to evoke action potentials. In simulations where the axonal diameter *D* (μm) was changed, the current amplitude was linearly adjusted with the diameter as *I*_inj_ = (*D*/2) × 100 pA to securely initiate spikes. To estimate the conduction velocity, we measured the travel time between nodes #40 and #90 by calculating the difference of the times at which the membrane potential reached its peak at these nodes. Then we divided the distance between the two nodes by the travel time to obtain the conduction velocity.

### Simulating unmyelinated axons

Unmyelinated axons were simulated as a series of excitable elements combined with the cable model (Koch, 1999). While a partial differential equation is used for the mathematical formulation, the simulated axon is actually divided into compartments when the propagation of an action potential is numerically calculated (Cooley and Dodge, 1966). Table 5 summarizes the equations and default parameters of the unmyelinated axon model. Similarly to the myelinated axon model, we used either the WB model (Table 1) or bEIF model (Table 3) for each compartment to simulate the spikes traveling along unmyelinated axons.

**Table 5.** Equations and parameters for unmyelinated axon models

**Variable**	**Equation**
Membrane potential *V*	$C_{m}\partial V(t,x)/\partial \text{t}=I_{\text{L}}+I_{\text{active}}+(\frac{D}{4{\text{R}}_{\text{ax}}})(\frac{\partial^{2}V}{\partial x^{2}})+I_{\text{inj}}$
Leak current	$I_{\text{L}}(x)={\text{G}}_{\text{L}}(E_{\text{L}}-V(t,x))$
Active current (for WB model)	$I_{\text{active}}(x)=I_{\text{Na}}(x)+I_{\text{K}}(x)$
Active current (for bEIF model)	$I_{\text{active}}(x)=I_{\text{dep}}(x)+I_{\text{rep}}(x)$
Intracellularly injected current	$I_{\text{inj}}(x)=0$ (default)
**Parameter**	**Value**
Axon diameter *D*	10 μm
Axial resistivity R_ax_	100 Ω cm

Parameters not listed in this table are unchanged from the single-compartment models (Tables 1, 3).

We simulated 301 compartments along a one-dimensional (non-branching) axon. The length of each compartment was set to 20 μm, which is sufficiently small compared to the length constant λ = 1.1 mm of this model axon (calculated as λ^2^ = *D*/(4G_L_R_ax_) = 1.25 mm^2^). Stimulus currents (amplitude *I*_inj_ = 10 nA, duration T = 1 ms) were injected intracellularly into the compartment #50 to evoke action potentials. In simulations where the axonal diameter *D* (μm) was changed, the current amplitude was linearly adjusted with the diameter as *I*_inj_ = (*D*/10) × 10 nA, to securely initiate spikes. To estimate the conduction velocity, we measured the travel time between nodes #100 and #200 by calculating the difference of the times at which the membrane potential reached its peak at these nodes. Then we divided the distance between the two nodes by the travel time to obtain the conduction velocity.

### Simulating extracellular stimulation

Extracellular stimulation of myelinated nerves can be simulated similarly to intracellular stimulation by introducing an additional variable of extracellular potential *U*_ex_ (Table 6), which is inversely proportional to the distance *r* from the current source (i.e., dimensionless, point electrode) as: *U*_ex_ = *ρ*_ex_*I*_ex_/(4π*r*), with *ρ*_ex_ being the extracellular resistivity and *I*_ex_ the amount of injected current (Abzug et al., 1974; McNeal, 1976; Rattay, 1986). The intracellular axonal current *I*_axon_ is determined by the gradient of intracellular potential *U*_in_, which is the sum of the membrane potential *V* and the extracellular potential *U*_ex_ (Table 6). The equations for the active and passive membrane currents that depend on the membrane potential are unchanged from the case of intracellular stimulation (Table 4).

**Table 6.** Equations and parameters for extracellular stimulation of myelinated axon models

**Variable**	**Equation**
Membrane potential *V* (of j-th node)	$c_{m}^{j}\;\text{d}V_{j}(t)/\text{dt}=I_{\text{L}}^{j}+I_{\text{active}}^{j}+I_{\text{axon}}^{j}$
Axonal current	$I_{\text{axon}}^{j}={\text{g}}_{\text{axon}}^{j-1}(U_{\text{in}}^{j-1}-U_{\text{in}}^{j})+{\text{g}}_{\text{axon}}^{j}(U_{\text{in}}^{j+1}-U_{\text{in}}^{j})$
Intracellular potential *U*_in_ (at j-th node)	$U_{\text{in}}^{j}=V_{j}+U_{\text{ex}}^{j}$
Extracellular potential *U*_ex_ (at j-th node)	$U_{\text{ex}}^{j}=\frac{\rho_{\text{ex}}I_{\text{ex}}}{4\pi r_{j}}$
Distance between electrode and j-th node	*r_*j*_* (see legend)
Extracellularly injected current	$I_{\text{ex}}=0$ (default)
**Parameter**	**Value**
Extracellular resistivity *ρ*_ex_	3.0 Ω m

Equations and parameters not listed in this table are unchanged from the case of intracellular current stimulation (Table 4). Note that the distance *r_j_* between the electrode and the node is determined by the location of the extracellular stimulus electrode and the geometry of the axon.

We simulated 141 nodes along a one-dimensional, straight axon. To evoke action potentials, stimulus currents (amplitude *I*_ex_ = -1 mA, duration T = 0.1 ms) were injected into the extracellular space located 1 mm away from the node #20. We tested both the WB and the bEIF model.

### Simulating AN axons

As an application of the bEIF model, we simulated spike conduction along the central axon of the mammalian AN. The model equations are the same as those for the myelinated bEIF axon (Table 4). Here we consider AN fibers that are tuned to either low frequency (located in the apex of the cochlea) or high frequency (located in the base of the cochlea). Table 7 lists the parameters for the low- and high-frequency AN models, and Table 8 summarizes relevant anatomic and physiological data used for calibrating the models. Since no physiological data were available for AN axons, we used values measured in cell bodies of spiral ganglion (AN) neurons.

**Table 7.** Parameters for low- and high-frequency AN models

**Parameter**	**Low-frequency AN model**	**High-frequency AN model**
Leak conductance density G_L_	0.2 mS/cm^2^	0.4 mS/cm^2^
Threshold for spike-generating current *V*_T_	-50.0 mV	-50.0 mV
Axon diameter *D*	2.5 μm	2.5 μm
Nodal length *L_*n*_*	2.0 μm	2.0 μm
Internodal length *L_*i*_*	350 μm	450 μm

The following parameters are unchanged from the single-compartment bEIF model (Table 3): membrane capacitance density *C_m_*, leak reversal potential E_L_, slope factor of the spike-generating current *K*_T,_ ceiling factor of the spike-generating current *a*_t_, starting voltage of repolarization current *V*_rep_, time constant of repolarizing conductance *τ*_rep_, amplitude factor of repolarizing conductance *A*_rep_. The axial resistivity R_ax_ is unchanged from the myelinated axon model (Table 4).

**Table 8.** Anatomic and physiologic data used for calibrating the AN models

**Item**	**Value**	**Animal**	**Reference**
Membrane capacitance	10.14 ± 1.68 pF 9 ± 2 pF 6.0 ± 1.7 pF	Guinea pig Guinea pig Rat	Santos-Sacchi (1993) Szabó et al. (2002) Jagger and Housley (2002)
Membrane resistance	200–800 MΩ (all CFs) 499 ± 290 MΩ (all CFs)	Guinea pig Rat	Santos-Sacchi (1993) Jagger and Housley (2002)
Membrane resistance	474 ± 230 MΩ (low CF) 285 ± 215 MΩ (high CF)	Mouse culture	Adamson et al. (2002)
Resting potential	-67.3 ± 5.7 mV -62 ± 9 mV -61.1 ± 7.0 mV	Guinea pig Guinea pig Rat	Santos-Sacchi (1993) Szabó et al. (2002) Jagger and Housley (2002)
Axonal diameter	2–3 μm (all CFs)	Cat	Liberman and Oliver (1984)
Internodal length	200–500 μm (low CF) 300–600 μm (high CF)	Cat	Liberman and Oliver (1984)

Physiologic data were measured in the cell body of spiral ganglion neurons. Reported standard errors were converted into standard deviations. CF: characteristic frequency.

From the reported capacitance of 10 pF (Table 8) and a standard capacitance density of 1.0 μF/cm^2^, the effective surface area of the cell body is estimated as 1000 μm^2^. Using the leak conductance densities of 0.2 mS/cm^2^ (low frequency AN model) and 0.4 mS/cm^2^ (high-frequency AN model), the membrane resistance of a 1000 μm^2^ membrane patch is calculated as 500 MΩ (low frequency) and 250 MΩ (high frequency), matching the measured physiologic values of mammalian spiral ganglion neurons (Table 8). The adopted leak reversal potential of -65.3 mV was unchanged from the single-compartment bEIF model (Table 3), as it matched the measured resting potential (Table 8). The (soft) threshold *V*_T_ for the spike-generating current of the EIF model is not directly related to measured spike thresholds, because of the fundamental differences in their definitions. We selected the value of *V*_T_ to roughly mimic the recorded spike waveforms of ANs (Adamson et al., 2002).

Reported internodal lengths of cat AN axons (Liberman and Oliver, 1984) were larger in the high-frequency region than in the low-frequency region, while the diameters of axons were similar between these regions (summarized in Table 8). As there were no systematic measurements available for the length of the node of Ranvier, we simply assumed that both low- and high-frequency AN fibers share the same nodal length of 2 μm. Previous simulations showed that the effect of nodal length of conduction velocity is relatively minor (Arancibia-Cárcamo et al., 2017). We simulated 40 nodes along a one-dimensional (non-branching) axon. The total length of the modeled axon roughly corresponds to the length of the cat AN fiber innervating the cochlear nuclei (Ryugo and Rouiller, 1988). Stimulus currents (amplitude *I*_inj_ = 60 pA, duration T = 1 ms) were injected intracellularly to the node #1 to evoke action potentials. The conduction velocity of a propagating spike was calculated as the distance between nodes #10 and #30 divided by the travel time between these nodes.

### Simulation environment

Numerical integration of the model equations was performed with the explicit (forward) Euler method, in combination with the Crank-Nicolson method for axonal current propagation (Moore et al., 1978). The time step we used was fixed to 4 μs, unless otherwise stated. To obtain an f-I curve for the single-compartment models, we injected a step current of varied amplitudes with a duration of 1000 ms to calculate the output spike rate of the model. Code was implemented with MATLAB R2015b (MathWorks).

To evaluate the computational costs of calculating axonal spike conductions with different models, we computed the integration time of voltage traces of an axon with 141 nodes, using identical configurations of the modeled axon to those described above in Simulating myelinated axons. Each trial was 400 ms long, and we repeated the computation 50 times to obtain an average integration time. In addition to MATLAB, numerical algorithms were also implemented in D (Alexandrescu, 2010), which is compiled into native machine code and is expected to run faster. Simulations were conducted on a desktop computer (Dell 1398 Precision T1700) with a 64-bit Windows 7 Professional Operating System, Intel Xeon CPU E3-1270 1399 v3 (4 core, 3.5 GHz) and 16 GB memory.

### Code accessibility

MATLAB implementation of the models is publicly available online at https://github.com/pinkbox-models.

## Results

### Responses the bEIF model

The main goal of this study was to construct a simple model of spike conduction along the axon. To this end, we first modified the sEIF model by limiting the exponentially growing inward current (Fig. 1*C*) and introducing a repolarizing current after spikes (Fig. 1*D*; see Materials and Methods). With these modifications, the resulting single-compartment bEIF model showed spike waveforms that were more similar to those of the HH-type WB model than the sEIF model (Fig. 1*A*,*E*), while keeping its f-I relationship largely comparable to those of both the WB and sEIF models (Fig. 1*B*).

### Spike conduction in myelinated axons

**Figure 2.**
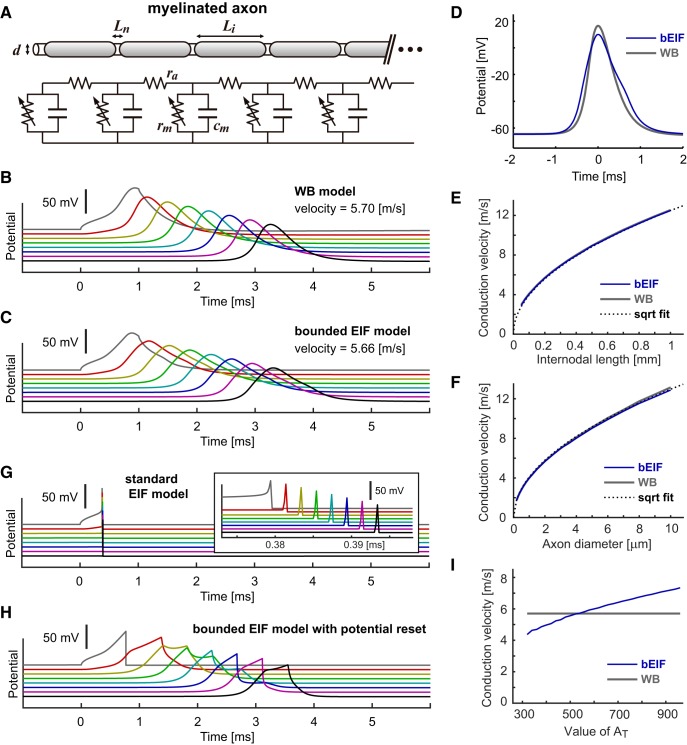
Response properties of myelinated axon models. ***A***, Schematic drawing (top) and modeled electric circuit (bottom) of a myelinated axon with a diameter *D*, nodal length *L_n_* and internodal length *L_i_*. Each nodal compartment has a capacitance *c_m_* and (voltage-dependent) resistance *r_m_* and is interconnected with neighboring nodes with an axial resistance *r_a_*. See Table 4 for the default parameter values. In panels ***B***, ***C***, ***G***, ***H***, voltage responses at every 10th node (i.e., each 2 mm apart) are shown. Currents were injected intracellularly into the node #20 (gray traces). ***B***, Spike conduction along the modeled myelinated axon simulated with the WB model. ***C***, Spike conduction along the modeled myelinated axon simulated with the bEIF model. ***D***, Shapes of conducted spikes of the bEIF and WB models. The peaks of both traces are aligned at time 0. ***E***, Dependence of simulated conduction velocity *u* (m/s) on the axon diameter *D* (μm). The dotted curve shows a square root fit by $u=4.1\sqrt{D}$. ***F***, Dependence of conduction velocity *u* (m/s) on the internodal length *L_i_* (μm). The dotted curve shows a square root fit by $u=0.395\sqrt{L_{i}}$. ***G***, Spike conduction along the modeled myelinated axon simulated with the sEIF model. Inset, Expanded traces around the spike generation. In panel ***G***, we used a time step of 0.2 μs to faithfully simulate the rapidly increasing membrane potentials. ***H***, Spike conduction along the modeled myelinated axon simulated with the bEIF model, with an instantaneous potential reset instead of the repolarizing current. ***I***, Dependence of conduction velocity *u* on the ceiling value *A*_T_ of the bEIF model (blue). Conduction velocity of the WB model (5.7 m/s) is also shown as a reference (thicker gray line). When necessary (typically for large and small values of *A*_T_), the starting voltage for repolarization current *V*_rep_ was readjusted (down to -20 mV from the default value of +15 mV using a step of 5 mV) to make sure the membrane potential returned to rest after spiking.

We simulated spike conduction along a myelinated axon by connecting excitable compartments with an axial resistance (Fig. 2*A*; Materials and Methods). The voltage dynamics of each compartment was simulated by either the WB or bEIF model. As in earlier studies with the HH model (FitzHugh, 1962; Brill et al., 1977; Rattay et al., 2003), stable propagation of an action potential was observed for the WB model (Fig. 2*B*). With the bEIF model, simulated spike propagation was also stable (Fig. 2*C*) and the estimated conduction velocity was comparable to that of the WB model. The relative insensitivity of the conduction velocity to the detailed spike-generating mechanisms was reported in an earlier study that compared the conductance-based HH model with the permeability-based Frankenhaeuser–Huxley model (Moore et al., 1978).

In contrast to the single-compartment setting (Fig. 1*E*), the simulated propagating spike waveform was wider for the bEIF model than for the WB model (Fig. 2*D*). This is because the repolarizing conductance of the bEIF model is static (i.e., independent of the additional axial current), whereas the ionic conductances in the WB model are more dynamically regulated by the membrane potential. The overall dependences of the conduction velocity on the internodal length (Fig. 2*E*) and the axonal diameter (Fig. 2*F*) were very similar between the WB and bEIF model, both well fitted by a square root curve. Assuming that the internodal length and the axonal diameter vary in proportion to each other (Hursh, 1939), the combined effect of these square root relationships results in the conduction velocity changing linearly with the size of the myelinated axon (Waxman, 1980).

Both the ceiling for the depolarizing conductance (Fig. 1*C*) and the after-spike repolarizing conductance in the bEIF model (Fig. 1*D*) are necessary for simulating stable spike propagation. Because of the exponential dependence of the depolarizing current on the membrane potential (Eq. 3), the membrane potential of the sEIF model quickly blows up to infinity once a spike is initiated (Touboul, 2009). Due to this instantaneous divergence of the membrane potential, the simulated action potential did not properly propagate along the axon when the sEIF model was used instead of the bEIF model (Fig. 2*G*). The ceiling of the depolarizing conductance in the bEIF model slows down the voltage change near the peak of the action potential, resulting in a more realistic spike shape and stable spike conduction than the sEIF model.

When the repolarization current of the bEIF model was replaced by an abrupt potential reset, propagation of electrical activity was observed, but the simulated waveforms were not uniform across the axonal compartments (Fig. 2*H*). This is because the potential reset (as in the sEIF model) is equivalent to injecting an enormous negative current within a small time step, which leads to discontinuous changes of the membrane potential. In contrast, the spike-mimicking repolarizing current in the bEIF model does not cause such discontinuous, unstable changes. As suggested by the simulation results for the sEIF model (Fig. 2*G*), the level of the depolarization ceiling factor (value of *A*_T_; Table 3; Fig. 1*C*) in the bEIF model affects the conduction velocity (Fig. 2*I*).

### Spike conduction in unmyelinated axons

**Figure 3.**
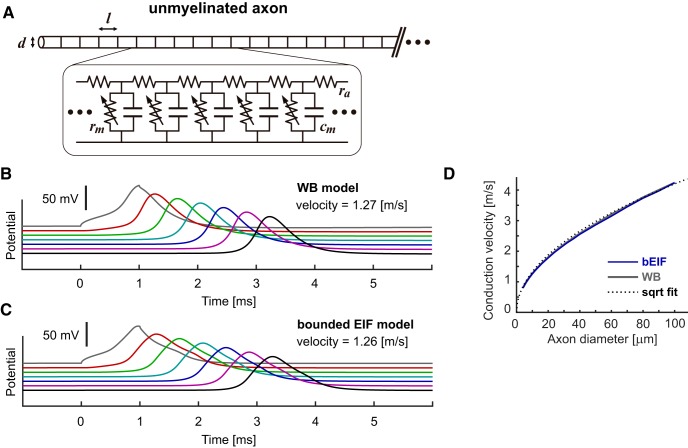
Response properties of unmyelinated axon models. ***A***, Schematic drawing (top) and modeled electric circuit (bottom) of an unmyelinated axon with a diameter *d.* Each nodal compartment of length *l* has a capacitance *c_m_* and (voltage-dependent) resistance *r_m_* and is interconnected with neighboring nodes with an axial resistance *r_a_*. See Table 5 for the default parameter values. In panels ***B***, ***C***, voltage responses at every 25th node (i.e., each 0.5 mm apart) are shown. Currents were injected intracellularly into the node #50 (gray traces). ***D***, Dependence of simulated conduction velocity *u* (m/s) on the axon diameter *D* (μm). The dotted curve shows a square root fit by $u=0.42\sqrt{D}$.

By connecting excitable units (Fig. 3*A*), spike conduction along an unmyelinated axon can also be simulated (Fig. 3*B*, for the WB model, Fig. 3*C*, for the bEIF model). As in myelinated axons, the simulated waveform was slightly wider for the bEIF model than for the WB model. Both models, however, showed similar conduction velocities. Furthermore, the dependence of the simulated conduction velocity on the axon diameter was largely comparable between these two models (Fig. 3*D*), displaying a square root relationship typical for unmyelinated axons (Waxman, 1980). These simulation results demonstrate that the bEIF model can be used for simulating propagation of action potentials in both myelinated and unmyelinated axons.

### Extracellular stimulation

**Figure 4.**
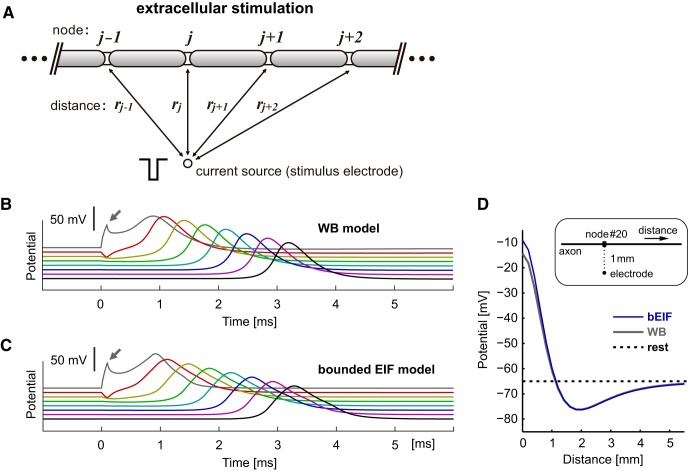
Responses of myelinated axon models to extracellular stimulation. ***A***, Schematic drawing of a myelinated axon stimulated with extracellular current injection. The extracellular voltage at each node is determined by the distance between the node and the stimulus electrode (see Materials and Methods for the equations). ***B***, Spike conduction along the modeled myelinated axon simulated with the WB model. ***C***, Spike conduction along the modeled myelinated axon simulated with the bEIF model. In panels ***B***, ***C***, voltage responses at every 10th node (i.e., each 2 mm apart) are shown. Gray arrows indicate the intracellular responses caused by the extracellular negative current injection. ***D***, Location-dependent voltage responses to extracellular stimulation. Simulated membrane potentials at the offset of extracellular current stimulation (-1 mA, 0.1 ms) are plotted as a function of the distance from the node #20 (gray traces in ***B***, ***C***), which is the closest node to the stimulus electrode, with a separation of 1 mm (see inset for a schematic drawing).

Neuroprosthetic devices usually use extracellular stimulation (Rattay et al., 2003). To test the applicability of the model to prosthetic stimulation, we simulated the responses of the modeled axon to extracellular current injection (Fig. 4*A*). In contrast to the case of intracellular stimulation, where the extracellular space was assumed to be isopotential, extracellular injection of current produces a gradient of extracellular potential, which is the source of the intracellular axial current along the modeled axon. For both the WB (Fig. 4*B*) and the bEIF (Fig. 4*C*) models, an extracellularly injected negative current induced positive responses at the closest node (Fig. 4*B*,*C*, small arrows) that lead to spike initiation. Depending on the relative location between the node and the electrode, the response of each node to the extracellular current is either depolarization or hyperpolarization (Fig. 4*D*), as was demonstrated in earlier modeling studies (Rattay, 1986; Warman et al., 1992). The overall responses, including spike generation and conduction, were largely similar between the two models (Fig. 4*B*,*C*), confirming that the bEIF model can be used not only with intracellular current injection, but also with extracellular stimulation.

### Application to ANs

**Figure 5.**
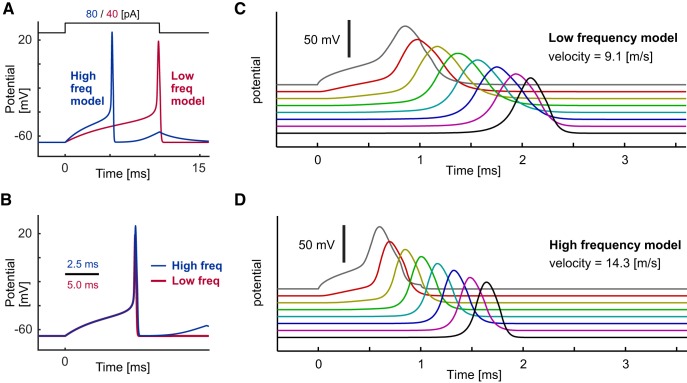
Response properties of AN axon models. See Table 7 for the default parameter values. ***A***, Spike responses of the low-frequency (red) and high-frequency (blue) single-compartment AN models driven by step current inputs. ***B***, Same traces as in ***A*** but with a rescaled time axis. ***C***, Spike conduction along the modeled low-frequency myelinated AN axon. ***D***, Spike conduction along the modeled high-frequency myelinated AN axon. In panels ***C***, ***D***, voltage responses at every five nodes are shown.

As an application of the bEIF model, we simulated the spike initiation and conduction of AN fibers. First, we constructed a single-compartment model of low- and high-frequency ANs (see Materials and Methods for details). Previous physiological measurements showed tonotopic (frequency-dependent) variations of membrane properties in spiral ganglion neurons (AN cells; see Rusznák and Szú́cs, 2009; Davis and Crozier, 2015 for reviews). The difference in input resistance between low- and high-frequency ANs was simply represented as the difference in the leak conductance density G_L_ of the bEIF model (Tables 7, 8), while other physiological parameters were identical between low- and high-frequency AN models. This modification led to a delayed spike initiation for the low-frequency model (Fig. 5*A*), although the overall voltage trajectory after scaling the time axis was largely indistinguishable between these two models (Fig. 5*B*). These simulation results are supported by physiological measurements reporting that low- and high-frequency neurons in mice had similar spiking thresholds but that the spike response latency was larger for low-frequency neurons (Adamson et al., 2002). As shown in Figure 1*B*, the bEIF (and WB) model expresses a Type I spiking behavior (i.e., zero spiking frequency at threshold), contrasting to the standard HH model that is Type II (non-zero spiking frequency at threshold). This Type I response property of the bEIF model enabled us to simulate the observed delayed spike generation in AN cells, which is generally inconceivable with Type II models.

Next, we adopted the same parameter sets to simulate spike conduction along the central part of myelinated AN fibers (Materials and Methods). The axonal diameter and internodal length were determined from previous anatomic measurements in cats (Liberman and Oliver, 1984). The simulated propagating spike waveform was wider for the low-frequency model (Fig. 5*C*) than for the high-frequency model (Fig. 5*D*), reflecting the difference in response latency (Fig. 5*A*). Calculated conduction velocities were 9.1 and 14.3 m/s for low- and high-frequency models, respectively. These values correspond to the measured velocities in cats (11.6 ± 1.6 m/s, Nguyen et al., 1999; ∼10 m/s, Miller et al., 2004). Our simulation results predict that high-frequency AN fibers should have higher conduction velocity than low-frequency fibers, because of the shorter response latency in the former. Testing this prediction will be a subject of future physiological studies in the field.

### Computational time

To compare the computational performances of the models, we calculated the average integration time for a modeled axon (Table 9). In agreement with the reduced number of equations and parameters, the bEIF model was several times faster than the WB model. In addition, the implementation with a compiled language led to a computation several times faster than the MATLAB code, while the computational advantage of the bEIF model was consistent between these implementations. These results roughly correspond to previous reports that compared the computational costs between HH-type and IF-type models (Destexhe, 1997; Ashida et al., 2015) and between C and MATLAB implementations (Goodman and Brette, 2008).

**Table 9.** Computational time for calculating axonal spike conduction

**Model**	**MATLAB**	**D (compiled into native code)**
WB	6.62 s	3.82 s
bEIF	2.36 s	1.09 s

A 400-ms membrane potential trace of an axon with 141 nodes was simulated (see Materials and Methods for more details). Average computational times of 50 trials are shown.

## Discussion

In this study, we introduced a simple model of nerve spike conduction based on the EIF model (Fig. 1). In comparison to the conventional HH-type model, our bEIF model has much fewer parameters (9 vs 25) and better computational performance (Table 9), but still retains fundamental functions for reproducing action potential propagation along the modeled axon (Figs. 2–4). Application of the model to ANs replicated measured conduction velocity in cats, and predicted that the velocity varies along the tonotopic axis (Fig. 5).

### Advantages of simple models

Simple phenomenological models can serve as a practical substitute for complex, conductance-based models, especially when descriptions of detailed ion channels dynamics are not required, when computational simplicity and mathematical transparency are desired, or when only insufficient empirical data are available for constraining a complex model. A simple neuron model was recently adopted, for instance, for a real-time simulation combined with an artificial fingertip (Oddo et al., 2016).

Fundamental lack of relevant biophysical data is a frequent impediment to the development of neural models for prosthetic simulation. To systematically tune the parameters of a neuron model (either IF or HH), measurements of intracellular membrane potentials are generally required (Badel et al., 2008; Rossant et al., 2010; Meliza et al., 2014). Because of ethical and technical limitations, however, measured data from human nerves are usually sparse (if not totally unavailable). Furthermore, a number of studies demonstrated that anatomical and physiological properties of human neurons may differ considerably from those of other animals (Felix, 2002; Angelino and Brenner, 2007; Eyal et al., 2016; Zhang et al., 2017). This makes it even more difficult to extrapolate non-human data to humans (see discussion below for related limitations in prosthetic simulations). Known as the “curse of dimensionality” (Almog and Korngreen, 2016), fitting model parameters of a nonlinear system becomes increasingly troublesome with an increase in the number of unconstrained parameters. Moreover, the geometry of “good” parameter sets may be highly skewed in the high-dimensional parameter space (Marder and Taylor, 2011), leading to a general difficulty in justifying the selection of parameters of complex models with a limited amount of data. The reduced number of parameters in the bEIF model may help mitigate these difficulties, at least when compared to complex HH-type models.

Because of its mathematical simplicity, the IF model and its variations have been used widely in theoretical and computational neuroscience (Koch, 1999; Gerstner et al., 2014, and references therein). Bifurcation analyses, for example, allow direct examination of spiking mechanisms of the EIF model (Touboul and Brette, 2008). Occasionally, IF-type models have also been used in multi-compartment simulations (Rospars and Lánský, 1993; Clopath et al., 2007; Saparov and Schwemmer, 2015; Aspart et al., 2016). However, these models normally have only one spike initiation site to avoid the problem with the instantaneous potential reset (see related discussion by Cessac and Viéville, 2008). In the bEIF model, the potential reset, which led to unstable waveforms of conducting spikes (Fig. 2*H*), was replaced with repolarizing conductance to replicate the downward trajectory of the spike waveform. Introduction of spike-mimicking current to IF-type models had already been suggested in prior studies (Ashida et al., 2015; Saparov and Schwemmer, 2015).

In early mathematical analyses of spike propagation, FitzHugh–Nagumo-type models were preferred, because they have fewer variables and are thus much easier to analyze than the HH model (Rinzel and Keller, 1973). The FitzHugh–Nagumo model, however, has major drawbacks: its fast activation variable (usually written as *V*) does not directly correspond to the membrane potential of a real neuron, and the parameters of the model have no clear biological interpretations. Our EIF model-based approach may be useful in alleviating these problems, as its parameters and variables have more intuitive biophysical meanings (e.g., membrane potential, conductances, spike-generating currents, etc.) while keeping a similar level of mathematical complexity as the FitzHugh–Nagumo model.

### Disadvantages and limitations

Previous studies have revealed a number of anatomical and physiological specializations in axons, which are nevertheless not always considered in existing axon models, including ours. For example, Nav1, Kv3, and Kv7 channels are clustered at the nodes of Ranvier, while Kv1 channels are distributed at juxtaparanodes under the myelin sheath (Debanne et al., 2011; Freeman et al., 2016; Kim and Rutherford, 2016). With some rare exceptions (McIntyre et al., 2002; Brown and Hamann, 2014), however, models of myelinated axons do not take the detailed distributions of ion channels into account. In our simulations (Fig. 2), spike-generating ionic currents were simply decomposed into depolarizing and repolarizing components without considering their actual ionic compositions. Moreover, histological studies suggested that ion channels are distributed unevenly along the actual AN fiber (Hossain et al., 2005; Yi et al., 2010; Kim and Rutherford, 2016). Hence our naive assumption that the axonal properties match the somatic properties (used in Fig. 5) is likely to be violated. Further refinement of the model would require detailed physiological characterization along each AN fiber and across the tonotopic axis.

The simulated voltage of the bEIF model does not undershoot after an action potential, since its repolarizing current is driven by the leak reversal potential E_L_. Introducing a different reversal potential (such as E_K_) could make the simulated spike waveform more realistic and closer to that of the WB model (Fig. 1*A*), but at the cost of adding another unconstrained parameter to tune. To calculate the repolarization conductance, we used an alpha function solely because of its simplicity, which nevertheless might have to be revised with a different function when a fine tuning of the depolarization phase is important. Moreover, spike shapes can significantly differ between the cell body and the axon (Kole et al., 2007). Further modifications and tuning of the model currents would thus be necessary to improve the physiological plausibility of simulated spikes propagating along the axon.

### Possible expansions and applications

To better account for the nonlinear membrane dynamics of a real neuron, a number of modifications of IF-type models have been proposed. Examples include bursting with T-type calcium current (Smith et al., 2000) or with persistent sodium current (Breen et al., 2003); spike-rate accommodation with slowly adapting current (Brette and Gerstner, 2005; Barranca et al., 2014) or with an after-hyperpolarization current (Zhou and Colburn, 2010; Barranca et al., 2014); subthreshold nonlinearity with low-voltage-activated potassium current (Svirskis and Rinzel, 2003; Ashida et al., 2015); and adaptation and stochastic fluctuation of the threshold (Takanen et al., 2016). Similar modifications can be incorporated into the bEIF model. We note, however, that the bEIF model would not fully replace HH-type models, but these two model types should rather complement each other. A user can and should choose an appropriate model according to the intended goals of modeling (Ashida et al., 2017): HH-type models for simulating the detailed ionic dynamics, and IF-type models for more phenomenological, handy description of neuronal spiking behavior.

Random opening of ion channels is suggested to affect neuronal coding properties (Ashida and Kubo, 2010; White et al., 2000; Negm and Bruce, 2014; Moezzi et al., 2016), including nerve conduction (Faisal and Laughlin, 2007). The utility of channel noise in cochlear implants has also been suggested (Rubinstein et al., 1999; White et al., 2000). To fully account for the ion channel stochasticity, Markov channel models in combination with an HH-type membrane equation would be required (Goldwyn and Shea-Brown, 2011). In practice, adding an adequate amount of artificial noise in a model can, at least in part, mimic the stochastic activity of electrically stimulated nerves (Joshi et al., 2017). Similarly, the addition of a noise term to the bEIF model would be necessary for simulating non-deterministic responses of the modeled nerve.

Earlier simulations of myelinated (FitzHugh, 1962; Brill et al., 1977; Moore et al., 1978) or unmyelinated (Cooley and Dodge, 1966) axons focused primarily on the biophysical mechanisms of nerve conduction. More recent modeling approaches aimed to gain clinical and engineering implications. Examination of degraded spike conductions caused by demyelination is one such example (Coggan et al., 2010; Brown and Hamann, 2014; Resnick et al., 2018). Moreover, driven by the rapid development of implantable devices that use electrical pulses to restore the functions of peripheral and central nerves (for reviews, see Masani and Popovic, 2011; Eiber et al., 2013), modeling approaches to simulate the activity patterns of electrically stimulated nerves have become an important tool for evaluating and predicting the performance of these prostheses (Rattay et al., 2003; also see Craver, 2010 for related philosophical considerations).

Recent prosthetic simulations using conductance-based (or other related) models include electrical stimulation of the retina (Barriga-Rivera et al., 2017), ANs (Briaire and Frijns, 2005; Negm and Bruce, 2014; O'Brien and Rubinstein, 2016; Joshi et al., 2017; Nogueira and Ashida, 2018), motor nerves (ElBasiouny and Mushahwar, 2007), and spinal and other peripheral nerves (Ladenbauer et al., 2010; Raspopovic et al., 2011; Capogrosso et al., 2013; Kent and Grill, 2013). A modeling approach in the cochlear implant study, for example, simulated several tens of thousands of excitable nodes distributed in three-dimensional space to predict the aggregated electrical response of the tissue (Nogueira et al., 2016). Such large-scale modeling studies generally require efficient phenomenological descriptions of neuronal spiking activity. Furthermore, as discussed above, simulations of human nerves often suffer from the lack of relevant physiological data. Our bEIF model thus offers a computationally efficient alternative to more complex (e.g., HH-type) models to be used in future prosthetic simulations.

10.1523/ENEURO.0112-18.2018.s01 Supplementary 1 Supplementary Matlab Code. Download Supplementary 1, ZIP file

## Synthesis

Reviewing Editor: William Stacey, University of Michigan Decisions are customarily a result of the Reviewing Editor and the peer reviewers coming together and discussing their recommendations until a consensus is reached. When revisions are invited, a fact-based synthesis statement explaining their decision and outlining what is needed to prepare a revision will be listed below. The following reviewer(s) agreed to reveal their identity: Ian Bruce.

The reviewers and I have discussed the paper and find it of potential interest. The main concern is that this model has a major limitation: it simulates intracellular current injection, where the actual physical scenario is extracellular current. The translation from the EC to IC current is not always straightforward. While there might be some simply kernel to simulate this, the true physiology is very dependent upon spatial location of channels, dendrites, etc. These are actually listed as two items in R2 (#2 and #3). The reviewers feel that while it would clearly be best to find a better way of modeling this, it might not be feasible for this particular submission. We would like for you to address this concern as best as you can. I would suggest a detailed discussion, but also some toy model demonstration or perhaps past literature to give an idea of how clinical stimulation might translate into these results. This is a difficult problem, and we would like this work to move the field along somewhat rather than ignoring it.

There are several minor concerns described below, which should also be addressed.
